# Elucidating Immune Cell Changes in Celiac Disease: Revealing New Insights from Spectral Flow Cytometry

**DOI:** 10.3390/ijms26072877

**Published:** 2025-03-21

**Authors:** Sara Gómez-Aguililla, Sergio Farrais, Carla Senosiain, Natalia López-Palacios, Beatriz Arau, Ángela Ruiz-Carnicer, Rebeca Sánchez-Domínguez, María Corzo, Isabel Casado, Mar Pujals, Andrés Bodas, Carolina Sousa, Concepción Núñez

**Affiliations:** 1 Laboratorio de Investigación en Genética de Enfermedades Complejas, Hospital Clínico San Carlos, Instituto de Investigación Sanitaria del Hospital Clínico San Carlos (IdISSC), 28040 Madrid, Spain; sgaguililla@salud.madrid.org (S.G.-A.); mcorzo@ucm.es (M.C.); 2 Servicio de Aparato Digestivo, Hospital Universitario Fundación Jiménez Díaz, IIS-Fundación Jiménez Díaz, 28040 Madrid, Spain; sfarraisv@quironsalud.es; 3 Departamento de Medicina, Universidad Autónoma, 28049 Madrid, Spain; 4 Servicio de Aparato Digestivo, Hospital Universitario Ramón y Cajal, 28034 Madrid, Spain; carla.senosiain@salud.madrid.org; 5 Servicio de Aparato Digestivo, Hospital Clínico San Carlos, Instituto de Investigación Sanitaria del Hospital Clínico San Carlos (IdISSC), 28040 Madrid, Spain; nlopezp@salud.madrid.org; 6 Department of Gastroenterology, Hospital Universitari Mutua Terrassa, 08221 Barcelona, Spain; beatrizarau@mutuaterrassa.es (B.A.); mpujals@mutuaterrassa.cat (M.P.); 7 Centro de Investigación Biomédica en Red de Enfermedades Hepáticas y Digestivas (CIBERehd), Instituto de Salud Carlos III, 28029 Madrid, Spain; 8 Departamento de Microbiología y Parasitología, Facultad de Farmacia, Universidad de Sevilla, 41012 Sevilla, Spain; acarnicer@us.es (Á.R.-C.); csoumar@us.es (C.S.); 9 División de Terapias Innovadoras, CIEMAT y Unidad de Terapias Avanzadas, IIS-Fundación Jiménez Díaz y Universidad Autónoma, 28040 Madrid, Spain; rebeca.sanchez@ciemat.es; 10 Centro de Investigación Biomédica en Enfermedades Raras (CIBERER), 28029 Madrid, Spain; 11 Servicio de Anatomía Patológica, Hospital Clínico San Carlos, Instituto de Investigación Sanitaria del Hopital Clínico San Carlos (IdISSC), 28040 Madrid, Spain; isabel.casadofa@salud.madrid.org; 12 Servicio de Pediatría, Hospital Clínico San Carlos, Instituto de Investigación Sanitaria del Hospital Clínico San Carlos (IdISSC), 28040 Madrid, Spain; andres.bodas@salud.madrid.org; 13 Redes de Investigación Cooperativa Orientada a Resultados en Salud (RICORS), 28029 Madrid, Spain

**Keywords:** gluten-free diet, gluten challenge, spectral cytometry, T cells, B cells

## Abstract

Celiac disease (CD) is an immune-mediated enteropathy of the small intestine triggered by gluten ingestion. Although the small bowel is the main organ affected, peripheral blood cell alterations have also been described in CD. We aimed to investigate immunological cell patterns in the blood of treated CD patients and in response to a 3-day gluten challenge (GC). Blood samples were collected from 10 patients with CD and 8 healthy controls on a gluten-free diet at baseline and 6 days after initiating the GC. All the samples were analyzed by spectral flow cytometry using a 34-marker panel. We found that patients with CD displayed a lower proportion of memory B cells compared to healthy controls, both at baseline and post-GC. Additionally, we observed the previously reported activated gut-homing CD4^+^, CD8^+^, and TCRγδ^+^ T lymphocytes on day 6 post-GC, and found the CD8^+^ subpopulation to be the most readily identifiable by flow cytometry. Importantly, the CCR9 marker proved effective in enhancing the selection of these gluten-responsive T cells, offering the potential for increased diagnostic accuracy. Spectral flow cytometry involves a complex data analysis, but it offers valuable insights into previously unexplored immunological responses and enables in-depth cell characterization.

## 1. Introduction

Celiac disease (CD) is an immune-mediated small intestinal enteropathy triggered by dietary gluten exposure in genetically predisposed individuals [1]. The small bowel is the main organ affected, but changes in peripheral blood also appear to be involved in CD pathogenesis. In treated CD patients, it is well known that gluten-specific CD4^+^ T cells are detectable in blood 6 days after the initiation of a 3-day gluten challenge (GC) [2]. These cells are accompanied by a wave of activated gut-homing CD8^+^ and γδ^+^ T cells [3,4], which are all key elements in the pathogenesis of the disease. Gluten-specific CD4^+^ T cells are considered the primary drivers of CD, as they recognize gluten peptides bound to HLA-DQ2/DQ8 receptors and initiate the immunological cascade leading to villous atrophy, largely mediated by CD8^+^ T cells in the intestinal epithelium. The increase in the γδ^+^ T cell intraepithelial subpopulation is also a hallmark of CD, although its role in the disease is not yet fully understood. The detection of these T cells in blood has important implications for diagnosing CD, developing novel therapeutic approaches, and identifying gluten-immunodominant epitopes, highlighting the importance of an in-depth characterization of all these cells [5,6,7]. In addition, other cell types have been found to be altered in the blood of patients with CD, with some changes persisting even after starting a gluten-free diet (GFD) [8,9,10].

For many decades, the phenotyping of peripheral blood cells has been performed to gain insights into CD pathology and identify new biomarkers or treatments [11,12]. Frisullo et al. reported a decrease in the percentage of circulating CD25^+^ FOXP3^+^ CD4^+^ regulatory T cells (T reg) when patients started a GFD [13]. In 2017, Cook et al. found that treated CD patients had fewer circulating total memory T reg cells compared to healthy controls (HC), but higher CD39^+^ memory T reg cells [8]. B lymphocytes have also been extensively studied, with changes in the proportion of memory or regulatory B cells observed in patients with CD (children and adults) compared to HC [9,14]. Additionally, circulating monocytes and dendritic cells have been thoroughly characterized in acute CD, potential CD, treated CD, and HC to assess cell proportions or gut-homing profiles [10,15]. 

In recent years, developments in the field of spectral flow cytometry have enabled the simultaneous and in-depth analysis of diverse cellular changes across multiple cell types within a single sample. The inclusion of numerous markers increases the likelihood of identifying unknown cellular phenotypes [16]. It must be noted that large panels of approximately 40 markers can be handled simultaneously. Advances in flow cytometry have been accompanied by the development of new analytical methods allowing for the identification of cell subpopulations that require the simultaneous consideration of multiple markers. Automated, unsupervised analyses significantly enhance the efficiency of and capacity for discovering novel cell populations by reducing the analysis time, minimizing subjectivity and bias, and improving reproducibility [17]. This makes spectral flow cytometry a powerful tool for understanding differences in the immune response between different groups of individuals by studying a wide range of markers, including those related to activation and homing. In the context of a 3-day GC, this technology may offer insights into how cell populations react to gluten re-exposure and unravel the mechanisms driving CD pathogenesis. 

We aimed to identify, by spectral flow cytometry, the differences in the immunological cell patterns between patients with CD and HC on a GFD and to explore changes in their cell responses on day 6 following a 3-day gluten reintroduction. We also attempted to improve the characterization of CD4^+^, CD8^+^, and TCRγδ^+^ T lymphocytes mobilized in the peripheral blood in response to a 3-day GC.

## 2. Results

A total of 10 patients with CD (women: 78%; mean age: 44.11 ± 5.61 years; time on a GFD: 30.22 ± 7.42 months) and 8 HC (women: 56%; mean age: 34.11 ± 4.09 years; time on a GFD: 1 ± 0 months) were included in the study.

**Figure 1:**
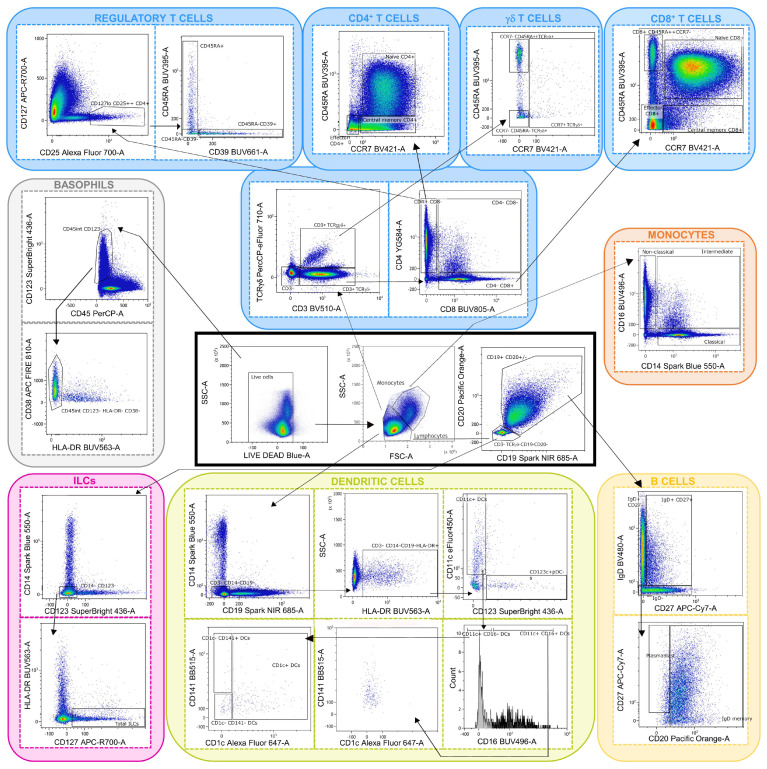
Flow cytometry gating strategy used to distinguish major immune cell populations, including CD4^+^ T cells, CD8^+^ T cells, TCRγδ^+^ T cells, B cells, monocytes, dendritic cells, basophils, and innate lymphoid cells (ILCs).

All the major immunological cell populations were accurately identified through manual gating and comprised CD4^+^, CD8^+^, and TCRγδ^+^ T lymphocytes; B lymphocytes; monocytes; dendritic cells; basophils; and innate lymphoid cells (Figure 1). Cell populations that needed the CD56 marker to be identified were excluded from the analysis due to technical issues relative to the expression of CD56 detected during the experiment. 

### 2.1. Immunological Cell Patterns

**Figure 2:**
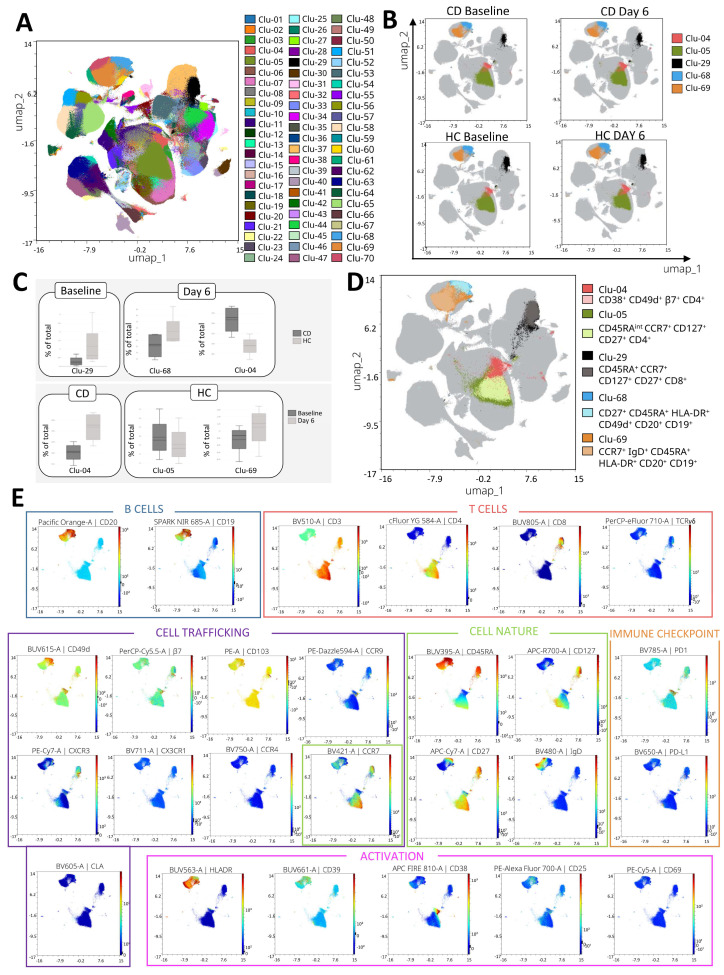
Unsupervised analysis of CD45^+^ cells. (**A**) Overlay of the 70 clusters generated by FlowSOM in the dimensionality reduction. (**B**) Clusters mapped by group and time point, highlighting the five significant clusters identified and their location in the dimensionality reduction. (**C**) Boxplots displaying significant comparisons (*p* < 0.05) between groups at each time point (CD: celiac disease; HC: healthy controls) and between time points (baseline vs. day 6) within each group. (**D**) Dimensionality reduction plot showing the overlap between the significant clusters identified by the unsupervised analyses and the manually gated populations. (**E**) Visualization of the significant clusters with their relative expression of cell lineage and phenotype markers. Lineage markers that were expressed and contributed to the identification of the representative population of each cluster are presented, along with all markers related to cell trafficking, cell nature, activation, and immune checkpoints.

The 70 clusters identified in the CD45^+^ subset using Uniform Manifold Approximation and Projection (UMAP) combined with Flow Cytometry Self-Organizing Mapping (Flow-SOM) are shown in Figure 2A. The distribution of the major cell types across the dimensionality reduction is included in Appendix A. After the edgeR and SAM algorithms, five significant clusters were identified from the analysis of CD45^+^ cells (Figure 2B), all corresponding to the lymphoid lineage. Two corresponded to differences between the groups (CD vs. HC): Clu-29 at baseline and Clu-68 on day 6. Two clusters, Clu-05 and Clu-69, corresponded to differences across time points within the HC group. Finally, Clu-04 showed differences between the groups on day 6 and within the CD group across time points.

The relative abundance of each cluster in the groups of interest, expressed as a percentage of the total CD45^+^ cells analyzed, is shown in Figure 2C. To characterize the major cell populations within each cluster, we performed manual gating based on the markers expressed by the majority of cells. The manually selected populations are shown in Figure 2D, overlaid with their corresponding clusters. Clu-29, Clu-68, Clu-05, and Clu-69 showed a great overlap. However, Clu-04 showed less overlap. This cluster included a previously described minority population [18]. The defining markers of this population were the only ones that showed little to no variation in expression, and we attributed the observed differences in Clu-04 to the presence of this specific subset. Figure 2E shows the significant clusters along with the gradient of marker expression across each one. 

**Figure 3:**
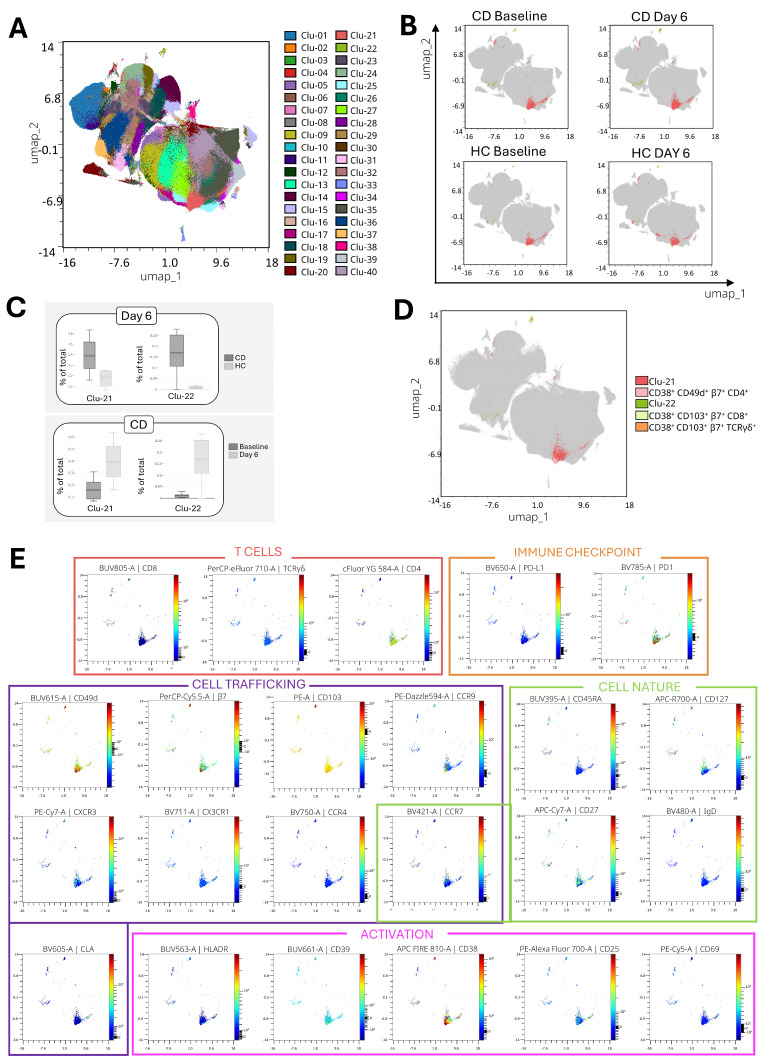
Unsupervised analysis of CD3^+^ cells. (**A**) Overlay of the 40 clusters generated by FlowSOM in the dimensionality reduction. (**B**) Clusters mapped by group and time point, highlighting the two significant clusters identified. (**C**) Boxplots showing significant comparisons (p < 0.05) between groups on day 6 (CD: celiac disease; HC: healthy controls) and between time points (baseline vs. day 6) in the CD group. (**D**) Dimensionality reduction showing the overlap between the significant clusters and the manually gated populations. (**E**) Visualization of the significant clusters with their relative expression of T cell lineage and phenotype markers.

Following the same analytical steps, the CD3^+^ analyses are presented in Figure 3. The distribution of the 40 clusters across the dimensionality reduction is shown in Figure 3A and the expression of the markers allowing for the identification of the representative populations is shown in Appendix A. Only two clusters, Clu-21 and Clu-22, showed significant differences (Figure 3B). These differences were observed when comparing baseline to day 6 samples within the CD group and when comparing CD vs. HC on day 6 (Figure 3C). The overlap between clusters and manually gated populations in the CD3^+^ analysis is shown in Figure 3D. Interestingly, the markers used for the manual selection of Clu-21 were the same as those used to manually identify Clu-04 in the CD45^+^ analysis. Concordantly, the manually gated population corresponding to Clu-21 showed less overlap and seemed to be the previously described minority population [18]. In contrast, the two populations identified in Clu-22 had a higher overlap in manual gating.

**Figure 4:**
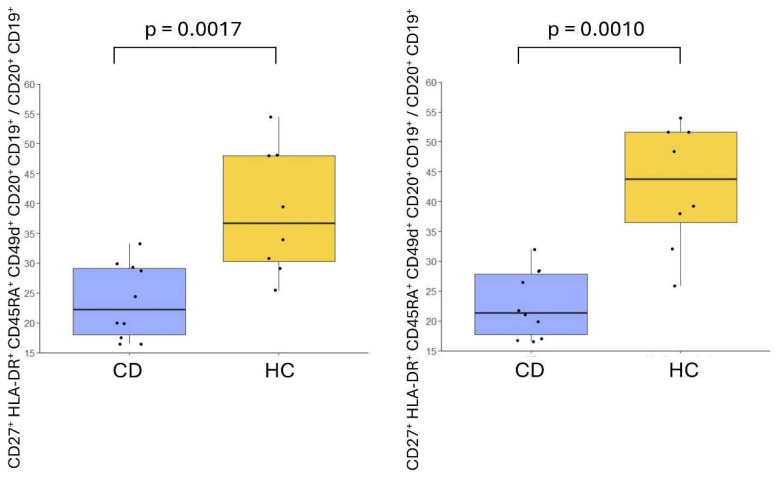
Percentage of the CD27^+^ HLA-DR^+^ CD45RA^+^ CD20^+^ CD19^+^ cells with respect to the total CD20^+^ CD19^+^ cells in patients with celiac disease (CD) and healthy controls (HC) on a gluten-free diet (baseline) and on day 6 following a 3-day gluten challenge.

Clu-21 corresponds to CD4^+^ T cells, while Clu-22 includes two lineage markers (CD8^+^ and TCRγδ^+^). Both clusters share the expression of CD49d, β7, CCR9, and CXCR3, which are all associated with intestinal trafficking, as well as the activation marker CD38. All the populations were characterized by the absence of PD-L1 (immune checkpoint marker), the chemokine receptors CCR4 and CX3CR1, CD69 (early activation/tissue residency), and CLA (associated with skin homing). Each cluster primarily comprised cell populations previously identified in patients with CD on day 6 following a 3-day GC (Figure 4) [4,5]. Specifically, CD103 and HLA-DR either showed reduced expression or were completely absent in Clu-21, which predominantly consisted of memory CD4^+^ T cells homing to the lamina propria, as indicated by the expression patterns of CD45RA^−^ CCR7^−^ and CD49d^+^ β7^hi^ CD103^−^, respectively. In contrast, Clu-22 also expressed β7 and CD103, indicating their migration to the intestinal epithelium, along with the activation marker HLA-DR. These clusters differed in their level of expression of CCR7 and the immune checkpoint marker PD-1, with CCR7 being almost absent in Clu-21 and PD-1 being highly expressed in this cluster (Figure 3E).

Table 1 summarizes the markers used for manual gating in each analysis. 

Thus, validation by manual gating confirmed four clusters with significant differences between groups or time points, with two clusters corresponding to each cell subset (Clu-68 and Clu-04 in the CD45^+^ approach and Clu-21 and Clu-22 in the CD3^+^ approach). As noted earlier, Clu-04 from CD45^+^ and Clu-21 from CD3^+^ exhibited the same marker expression, and we concluded that they identified the same cell subset. Therefore, we considered that the three unique clusters represented true differences between the analyzed groups or time points. These three clusters corresponded to four different populations (CD20^+^ CD19^+^, CD4^+^, CD8^+^, and TCRγδ^+^ T cells), as referred to hereafter.

The differences between patients with CD and HC in CD20^+^ CD19^+^ were confirmed at both time points when the CD27^+^ CD45RA^+^ HLA-DR^+^ CD49d^+^ CD19^+^ CD20^+^ cell population was manually gated. The percentage of CD27^+^ CD45RA^+^ HLA-DR^+^ CD49d^+^ CD20^+^ CD19^+^ cells relative to the total CD20^+^ CD19^+^ cells was significantly lower in CD. At baseline, this percentage was 23.57 ± 1.99% in patients with CD vs. 38.64 ± 3.73% in HC (*p* = 0.0017), and on day 6, it was 22.8 ± 1.77% in patients with CD vs. 39.2 ± 4% in HC (*p* = 0.0010). These results are shown in Figure 4. This population corresponded to B lymphocytes with the memory phenotype (CD27^+^). A detailed examination of markers with variable expressions such as CD39, to evaluate activation or regulatory functions, or IgD, to classify into class-switched and non-class-switched memory B cells, did not provide additional insights to increase the differences between groups. Moreover, excluding the HLA-DR selection did not alter the results, as this marker is fully represented on B lymphocytes. No differences among the proportion of total B lymphocytes were found. 

**Figure 5:**
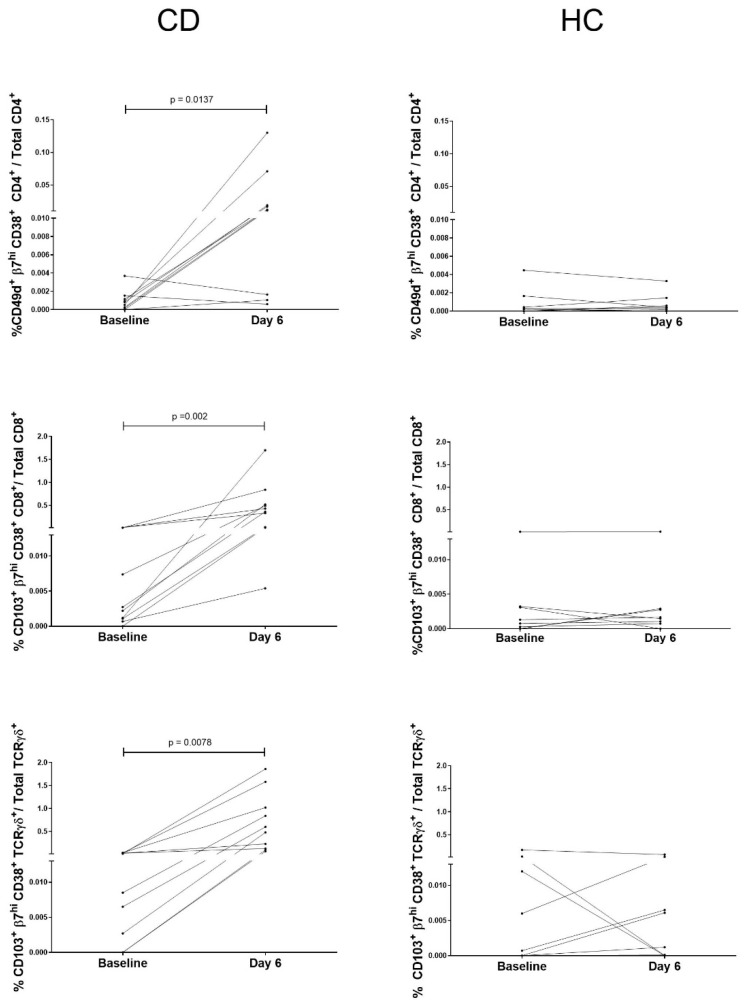
Percentage of activated gut-homing T cells in relation to the total CD4^+^, CD8^+^, and TCRγδ^+^ corresponding to patients with celiac disease (CD) and healthy controls (HC) on a gluten-free diet (baseline) and on day 6 following a 3-day gluten challenge. Only significant *p*-values are shown.

The predominant cell populations in Clu-04 in the CD45^+^ analysis and Clu-21 and Clu-22 in the CD3^+^ analysis have been previously described in patients with CD on day 6 following a 3-day GC [3,4,5]. Hereafter, they are referred to as activated gut-homing T cells. Consistently with previous reports, we observed significant differences between the baseline and day 6 within the CD group, as well as between patients with CD and HC on day 6 when they are manually gated (Figure 5). 

**Figure 6:**
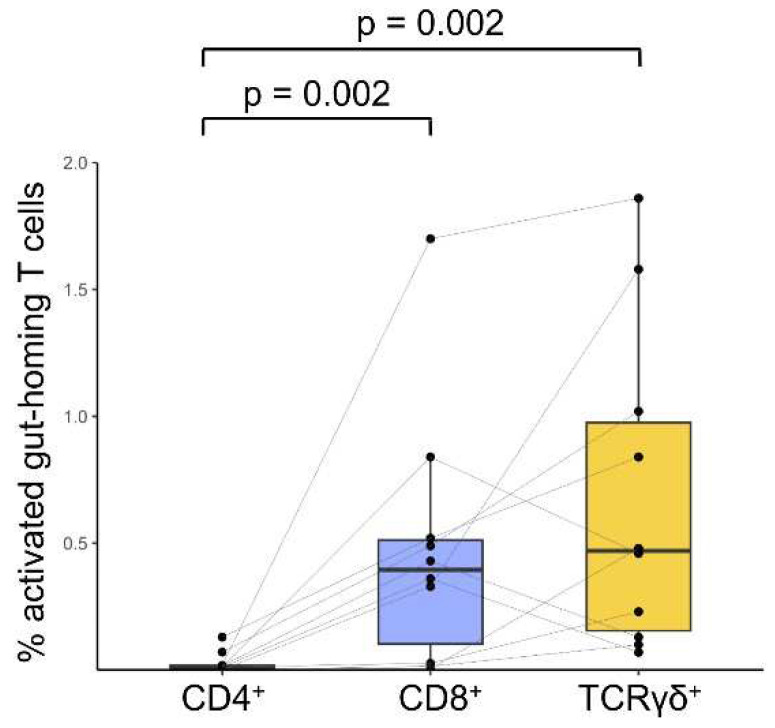
Percentage of the activated gut-homing T cells of interest across the three T cell subsets analyzed: CD4^+^, CD8^+^, and TCRγδ^+^ T cells. Only significant *p*-values are shown.

The markers used to identify these cell populations in peripheral blood were previously defined as β7^hi^ CD103^+^ CD38^+^ for CD8^+^ and TCRγδ^+^ T cells [2,3,5], and we used β7^+^ CD49d^+^ CD38^+^ for CD4^+^ T cells [18]. Using these gating strategies, we confirmed significant differences. The percentage of β7^+^ CD103^+^ CD38^+^ CD8^+^ T cells relative to the total CD8^+^ T cell population was significantly higher on day 6 compared to baseline in patients with CD (*p* = 0.002), as well as when comparing CD and HC participants on day 6 (*p* < 0.0001). Identical patterns were observed in β7^+^ CD103^+^ CD38^+^ TCRγδ^+^ and β7^+^ CD49d^+^ CD38^+^ CD4^+^ T cells: baseline vs. day 6 in patients with CD (*p* = 0.009 and *p* = 0.0137, respectively), as well as between patients with CD and HC on day 6 (*p* < 0.0001 and *p* = 0.001, respectively). While all T cell subsets exhibited an increase in the percentage of activated gut-homing T cells, this effect was most pronounced in the CD8⁺ and TCRγδ ^+^ subsets (Figure 6).

The differences between patients with CD and HC in the four cell populations considered did not seem to be influenced by sex or age (Appendix B).

### 2.2. Characterization of CD4^+^, CD8^+^, and TCRγδ^+^ T Lymphocytes

**Figure 7:**
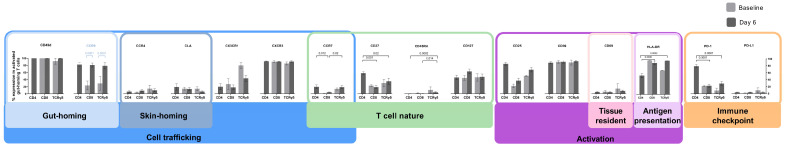
Mean percentage and standard error of the mean of phenotypic marker expression in manually identified activated gut-homing CD4^+^, CD8^+^, and TCRγδ^+^ T cells. Only significant *p*-values are shown, with *p*-values for differences between days displayed in blue and *p*-values for differences between cell types shown in black.

To improve the characterization of gut-homing T cells, we manually examined the variability in the expression of phenotypic markers within CD4^+^, CD8^+^, and TCRγδ^+^ gluten-induced T cell populations. When at least 10 cells with this phenotype were detected, they were included in the analysis. The mean percentage of marker expression within each cell group is summarized in Figure 7. High expression levels of CXCR3, CCR9, CD49d, and CD39 were identified on day 6 in all three T cell populations. Some cells with the same markers as the studied CD8^+^ and TCRγδ^+^ T cell populations were observed at baseline in patients with CD, but the percentage of CCR9 expression was significantly lower compared to that on day 6 in those cells. In the HC group, only one participant had at least 10 cells in each T cell subset, which was deemed sufficient for proper characterization. Elevated PD-1 expression was found only in CD4^+^ T cells. In contrast, CD8^+^ and TCRγδ^+^ cells presented significantly higher expressions of HLA-DR compared to CD4^+^ cells. Intermediate values differing between the groups were observed in several markers related to the T cell nature. CD27 showed a higher expression in the CD4^+^ subset. CD45RA and CCR7 exhibited a low expression across the three T cell populations, and slight, but significant, differences were observed between them.

## 3. Discussion

In this study, we used spectral flow cytometry to examine peripheral blood cell populations in individuals with CD compared to HC, considering two conditions: at baseline on a GFD and on day 6 following a 3-day GC. The inclusion of a group of healthy volunteers following a GFD instead of a regular diet represents a novel aspect of this study. Our panel of markers was designed for the broad screening of major blood cell populations with a detailed focus on T cell subsets.

One of our key findings was a marked alteration in the proportion of memory, as indicated by the CD27^+^ expression, B cell subpopulations. We observed that patients with CD had a significantly lower proportion of memory B cells compared to HC participants, a result previously observed in treated pediatric CD patients [9]. In our control group, the memory and naïve B cell levels aligned with those seen in the general population, typically in the range of 58–79% and 14–40%, respectively [19,20]. Interestingly, when assessing total B lymphocytes, no significant differences emerged between the CD and HC groups, suggesting that patients with CD have a higher proportion of circulating naïve B cells. These findings are concordant with previous studies reporting elevated naïve B cell frequencies in other autoimmune conditions, such as multiple sclerosis, even after treatment [21]. Our flow cytometry panel was not initially designed for an in-depth characterization of B cells, but the finding of a reduced memory B cell population in patients with CD could hold significant relevance, potentially serving as a biomarker or contributing to a deeper understanding of immunopathology. Further analyses are needed to better characterize this population and help elucidate why it is diminished in patients with CD. Additionally, the inclusion of new markers may be helpful in evaluating the potential of this cell population as a biomarker once a GFD is initiated. It would also be valuable to include patients who have been on a GFD for an extended period of time to determine whether this pattern is restored and may reflect a completely normalized immune response.

The second significant result was the activation and proliferation of gut-homing CD4^+^, CD8^+^, and TCRγδ^+^ T cells in patients with CD following a 3-day GC, which has already been well established [3,4,18]. The CD4^+^ population is commonly studied using HLA-DQ2.5: gluten tetramers, allowing for the precise identification of gluten-specific cells. However, this method is technically complex and demands a large blood volume, which can limit its practicality, especially outside of research settings. Consequently, for certain applications, a simpler approach employing an alternative technology would be advantageous. In 2022, Christophersen et al. proposed that CD-driving pathogenic CD4^+^ T cells in the gut could be isolated using flow cytometry based on distinct phenotypic markers. This method not only simplifies the technological process, but also increases the number of specific cells selected by eliminating the need for disease-driving antigens [22]. Consistent with previous reports, our results suggest that this approach may be similarly applied to blood samples [3,4,5], and it may support the validity of flow cytometry in studying activated gut-homing CD8^+^ T cells on day 6 following a 3-day gluten challenge for diagnostic purposes, even though they are not considered gluten-specific cells. In addition, TCRγδ^+^ T cells can be selected; while previously discouraged from being used for CD diagnosis due to their low numbers in the blood [4], their inclusion may provide new insights into CD pathogenesis.

Previous studies have attempted to phenotype T cells on day 6 following a 3-day GC. Initially, HLA-DQ2.5 gluten tetramer-positive CD4^+^ T cells were characterized by a gut-homing (integrin β7^+^), activated (CD38^+^), and effector memory (CD45RA^−^ CCR7^low^ CD27^−^ CD62L^−^) phenotype. A similar profile was described in CD8^+^ and TCRγδ^+^ T cells [3]. More recently, building on the markers identified for gluten-specific CD4^+^ T cells, comparisons with CD8^+^ and TCRγδ^+^ T cells revealed that all three populations also consistently expressed the gut-homing markers CCR9, CXCR3, and CD49d, identifying these markers as hallmarks of gluten-reactive T cells [18]. Our present results confirm these earlier findings, with slight variations among markers for gluten-responsive CD4^+^ T cells compared to those for CD8^+^ and TCRγδ^+^ T cells. These variations reflect the clustering observed, where CD4^+^ T cells formed one distinct cluster, while CD8^+^ and TCRγδ^+^ T cells clustered separately (Figure 3D). According to our present data, CD4^+^ T cells can be properly characterized by β7^hi^ CD49d^+^ CD38^+^ CD39^+^ CCR9^+^ CXCR3^+^ PD-1^+^CD45RA^−^ CCR4^−^, while β7^hi^ CD49d^+^ CD103^+^ CD38^+^ CD39^+^ CCR9^+^ CXCR3^+^ HLA^−^DR^+^ CD45RA^−^ CCR4^−^ can be used for defining CD8^+^ and TCRγδ^+^ T cells. Notably, PD-1 was fully expressed only in CD4^+^ T cells, while the HLA-DR expression was higher in CD8^+^ and TCRγδ^+^ cells. Additionally, CD103 was expressed solely in the CD8^+^ and TCRγδ^+^ subsets, consistent with the localization of these subpopulations in the intestinal epithelium, in contrast with CD4^+^ T cells, which only express CD49d due to their lamina propria location. Slight differences in the expression levels of CD27, CD45RA, and CCR7 among the three T cell subsets were also observed, but their overall low expression is indicative of an effector memory phenotype. However, it is essential to distinguish between markers that define subpopulations and those valuable for diagnostic purposes, as some markers are redundant and do not enhance diagnostic specificity. In this context, CCR9 stands out as a key marker. Notably, we observed distinct patterns of CCR9 expression between patients with CD and non-CD controls and even between patients with CD at baseline and on day 6 following the 3-day GC, underscoring its utility in increasing diagnostic specificity [23]. Considering that some patients start a GFD on their own without prior evaluation, improving specificity is particularly important. Conversely, CD62L^−^, previously associated with an effector memory phenotype in these T cell subsets, was excluded from our panel, as its role is already well established and it does not add diagnostic value.

While our previous studies [4,5] demonstrated a very high sensitivity and specificity of the CD8^+^ T cell subset for CD diagnosis on a GFD, it can be hypothesized that analyzing the CD4^+^, CD8^+^, and TCRγδ^+^ T cell subsets simultaneously may further enhance the diagnostic accuracy. However, our present findings reveal that CD4^+^ T cell responses are significantly lower than those observed for CD8^+^ and TCRγδ^+^ T cells, which exhibit comparable proportions. This disparity in the response magnitude may be diagnostically relevant and discourage the use of CD4^+^ T cells for diagnostic purposes using this approach, despite being the gluten-specific cell subset. TCRγδ^+^ cells have previously been noted to have limited utility in diagnostics due to their low abundance in blood [4].

Beyond the diagnostic utility, the induction of these three T cell subsets following a short gluten challenge in patients with CD could also be beneficial for other clinical approaches. Specifically, the absence of these T cell responses in patients with CD following a 3-day GC may prove useful for selecting genetically modified, low-immunogenic cereals. Additionally, this lack of response could serve to evaluate the efficacy of novel biological therapies for CD.

In addition to the four cell populations described in this work, we cannot rule out the possibility that other differences between groups or time points exist, but were not detected due to the relatively small sample size.

Overall, our present findings highlight the utility of spectral flow cytometry, combined with both unsupervised and supervised analyses, in elucidating immune profiles associated with CD. However, an unsupervised analysis may follow various approaches depending on the primary research objective. Notably, limitations imposed by software tools on the data volume in dimensionality-reduction techniques suggest the employment of strategic sample inclusion to effectively capture shifts in minority cell populations. To avoid a reduction in the number of cells analyzed, it is advisable to include fewer samples when aiming to identify substantial changes in these cell populations. In such cases, the changes detected must be sufficiently pronounced, as exemplified by the activated gut-homing T cells reported here, which are largely absent in healthy controls and patients with CD on a GFD, but emerge in individuals with CD following a 3-day GC. Conversely, to detect more subtle differences, a larger variety of samples should be included, even if this requires reducing the number of data points per sample. This trade-off facilitates a broader exploration of potential variations.

In conclusion, spectral flow cytometry provides a powerful platform for acquiring extensive multidimensional data, though the management and interpretation of these complex datasets remains an area that continues to evolve and improve. To harness the full potential of this technology, employing unsupervised analytical methods is essential for reducing data complexity and generating preliminary insights, which can subsequently be validated with established analytical approaches. In this study, the use of spectral flow cytometry enabled the observation of distinct variations in B and T cell populations, providing valuable insights into the immune landscape of CD. Further investigation is warranted to explore the implications of these observations for CD management and potential treatment strategies.

## 4. Materials and Methods

### 4.1. Study Design

We conducted a multicenter, prospective, quasi-experimental clinical study at four tertiary centers in Spain: Hospital Universitari Mutua Terrassa (Barcelona), Hospital Clínico San Carlos, Hospital Universitario Fundación Jiménez Díaz, and Hospital Universitario Ramón y Cajal (Madrid).

### 4.2. Participants, Intervention, and Sample Collection

A total of 18 individuals were recruited: 10 patients with CD and 8 HC. All the participants underwent a 3-day GC as previously described [23]. Briefly, 10 g of low FODMAP powdered gluten (El Granero Integral™; Biogran S.L.; Madrid, Spain) was administered daily in a lactose-free liquid yogurt in the morning from day 1 to day 3. For each subject, two peripheral blood samples were collected in EDTA tubes: the first before gluten reintroduction (baseline) and the second after 6 days (day 6). A total of 36 blood samples were analyzed by flow cytometry.

### 4.3. Panel Design

A 35-marker panel was designed to identify and phenotype the major peripheral blood cell populations, including markers for lineage, homing, activation, and inhibition. It was adapted from the OMIP-69 panel [24], with 14 markers from the original design being omitted and 9 new markers introduced: CLA, PDL-1, CX3CR1, CCR4, integrin β7, CD103, CCR9, CD69, and CD49d (Appendix A, Table A1).

### 4.4. Sample Processing

The starting material consisted of 2 mL of whole peripheral blood. Erythrocytes were lysed using 1× lysis buffer at a 1:20 ratio for 15 min and removed by centrifugation. The remaining white blood cells were then processed following the OMIP-69 protocol [17]. Briefly, the LIVE/DEAD™ Fixable Blue Stain Fluorescence Viability reagent was added to fresh cells, and the cells were incubated for 15 min. After centrifugation, a staining buffer and monocyte blocker were added to reduce non-specific monocyte staining and fluorescence interactions, and the cells were incubated for 10 min. Subsequently, staining was performed in sequential steps as previously described to prevent marker interactions [24], for a total incubation time of 50 min. Finally, the cells were washed and resuspended in 800 µL of phosphate-buffered saline supplemented with 1% fetal bovine serum. All the reagents used in the protocol are listed in detail in Appendix A (Table A1). The samples were acquired using a Cytek™ Aurora instrument with 5 lasers. 

### 4.5. Quality Control and Reproducibility: Panel Validation

A gating strategy was carried out on all flow cytometry standard (FCS) files to identify the major cell populations and to confirm the quality and reproducibility of the panel being studied (Figure 1). When necessary, an unmixing correction was previously performed to reduce potential bias. These steps were carried out with the FlowJo™ v10 (BD Life Sciences) and OMIQ softwares. 

### 4.6. Unsupervised Analysis

An automated cleaning algorithm, FlowAI, was applied to each FCS file to retain only those high-quality events that passed the filter. UMAP and FlowSOM were used for a dimensionality reduction and the clustering of events, respectively. These algorithms were run on previously gated CD45^+^ singlets. Additionally, UMAP and FlowSOM were applied to previously identified CD3^+^ lymphocytes to detect subtle differences. Seventy clusters were predefined for the singlets and 40 for the T cells. The resulting clusters were further analyzed using the edgeR and SAM algorithms to identify differences between groups or across time points (baseline and day 6), respectively. Clusters showing significant differences were plotted to determine the corresponding cell populations and their specific markers. Unsupervised analyses were performed using the OMIQ software. 

### 4.7. Supervised Analysis

Significant cell populations identified through the unsupervised analysis were manually gated using the FlowJo software. The Shapiro–Wilk test was used to assess the normality of the cell population frequency distributions. Differences between groups of patients and between baseline and day 6 samples were evaluated using an unpaired and paired Student’s *t*-test, respectively, for normally distributed variables. The Wilcoxon test was used to compare non-normally distributed variables between groups. *P*-values of ≤0.05 were considered statistically significant. The statistical analyses were carried out using GraphPad Prism v9. 

The FlowJo software was also used for the further characterization of the subsets β7^hi^ CD103^+^ CD38^+^ CD8^+^, β7^hi^ CD103^+^ CD38^+^ TCRγδ^+^; and CD49d^+^ β7^hi^ CD38^+^ CD4^+^.

## Figures and Tables

**Table 1:** Significant clusters identified through the unsupervised analysis and the markers used for their manual gating.

Analysis	Cluster	Lineage	Phenotypic Markers
CD45^+^	Clu-29	CD8^+^ CD3^+^	CD127^+^ CD27^+^ CD45RA^+^ CCR7^+^
	Clu-68	CD20^+^ CD19^+^	CD27^+^ CD45RA^+^ HLA-DR^+^ CD49d^+^
	Clu-04	CD4^+^ CD3^+^	CD38^+^ CD49d^+^ β7^+^
	Clu-05	CD4^+^ CD3^+^	CD127^+^ CD27^+^ CD45RA^+^ CCR7^+^
	Clu-69	CD20^+^ CD19^+^	CCR7^+^ CD45RA^+^ HLA-DR^+^ IgD^+^
CD3+	Clu-21	CD4^+^	CD38^+^ CD49d^+^ β7^+^
	Clu-22	CD8^+^	CD38^+^ CD103^+^ β7^+^

**Figure A1:**
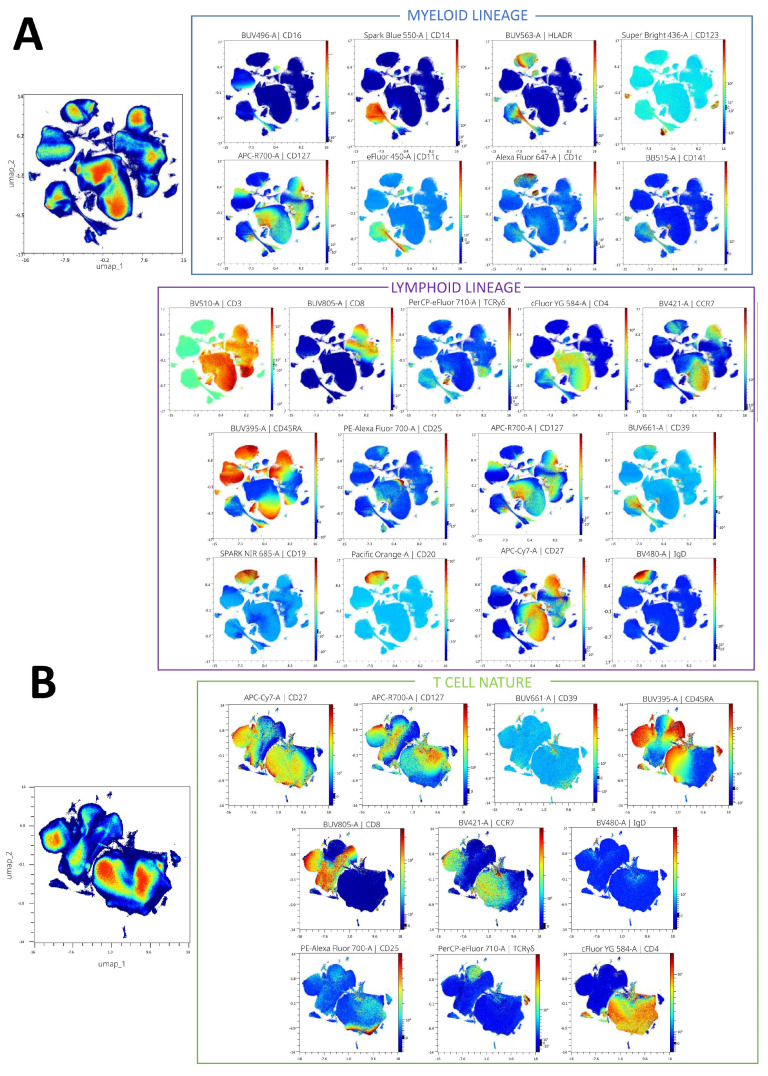
Density plots and marker expression gradients across the dimensionality reductions for (**A**) CD45^+^ and (**B**) CD3^+^ subsets.

**Figure A2:**
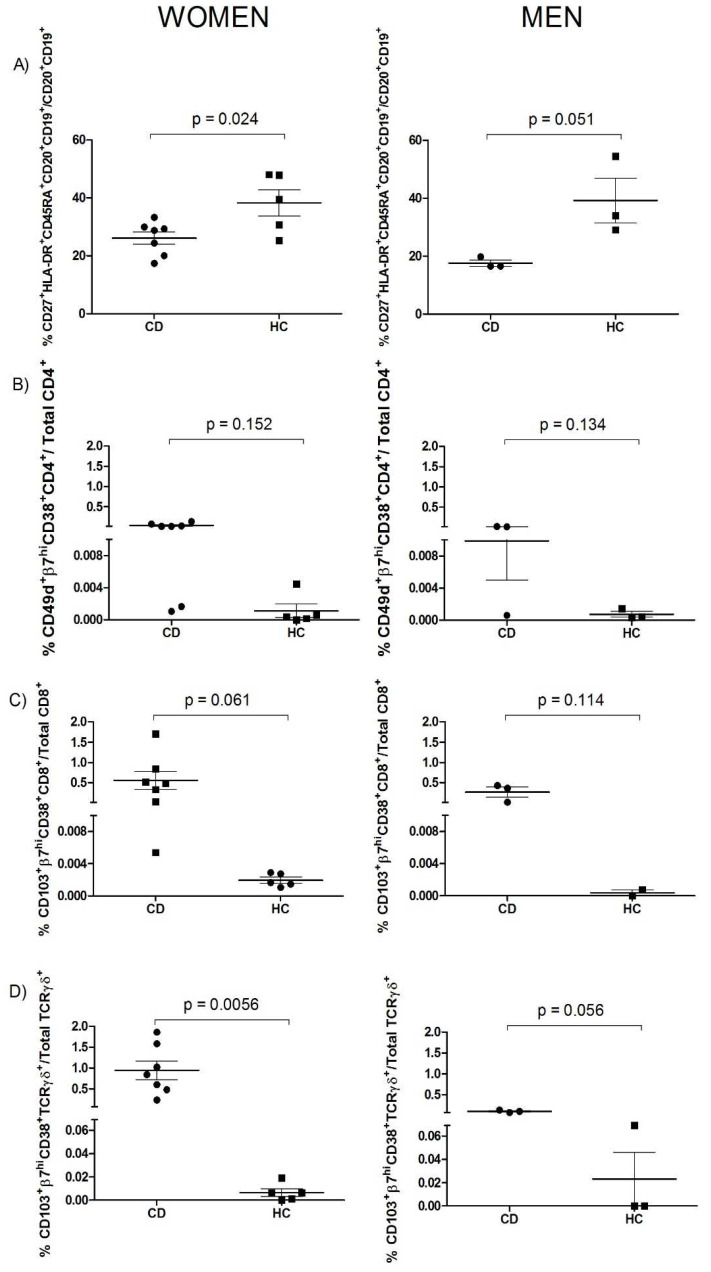
Graphical representation of the four cell subpopulations differentially expressed between patients with celiac disease (CD) and healthy controls (HC) stratified by sex. (**A**) CD27^+^ CD45RA^+^ HLA-DR^+^ CD19^+^ CD20^+^ in relation to the total CD19^+^ CD20^+^; (**B**) CD49d+β7^hi^ CD38^+^ CD4^+^ in relation to the total CD4^+^; (**C**) CD103+β7^hi^ CD38^+^ CD8^+^ in relation to the total CD8^+^; and (**D**) CD103+β7^hi^ CD38^+^ TCRγδ^+^ in relation to the total TCRγδ^+^. *P*-values were calculated using an unpaired Student’s *t*-test. Only significant *p*-values are shown.

**Figure A3:**
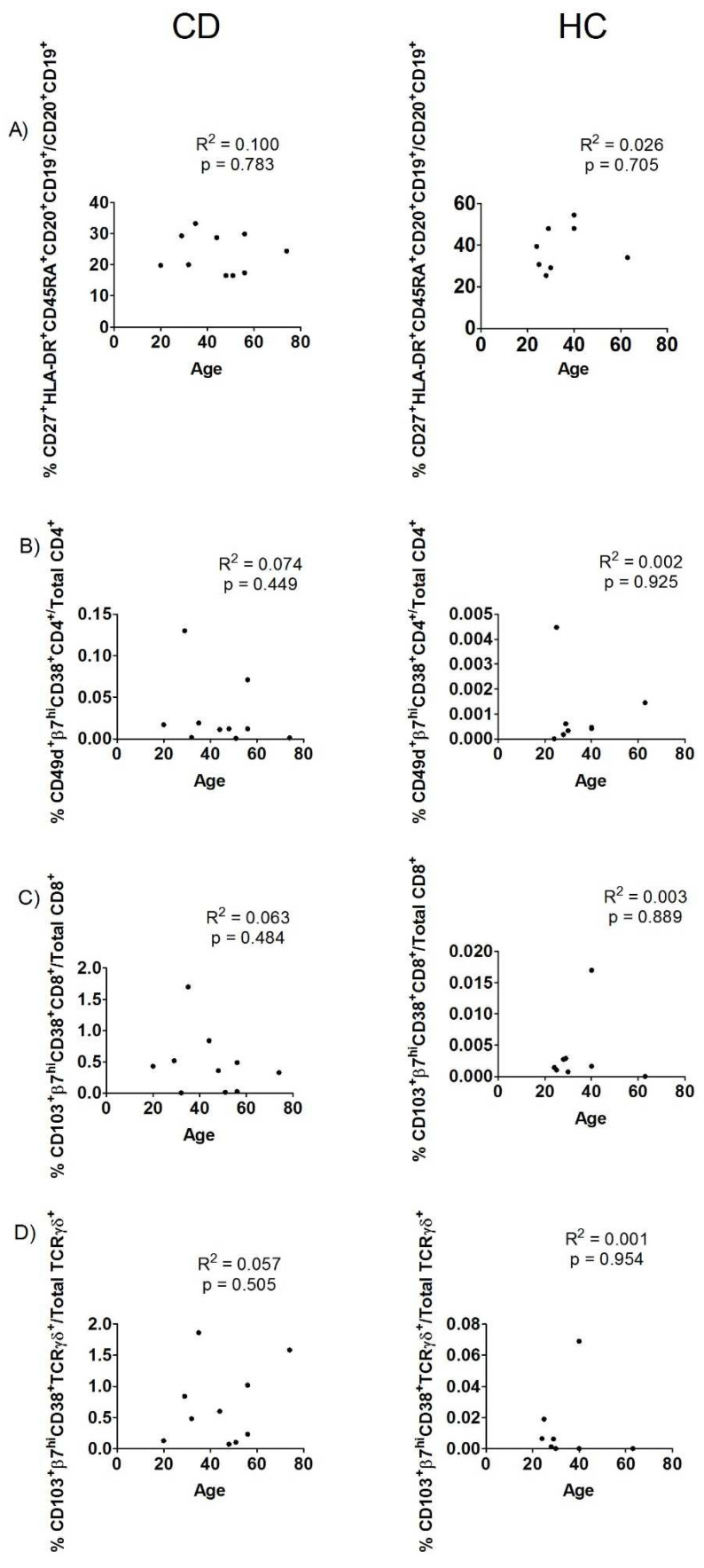
Correlation between age and the frequency of each differentially expressed cell subtype in samples collected on day 6 following the 3-day gluten challenge in celiac disease (CD) and healthy controls (HC). (**A**) CD27^+^ CD45RA^+^ HLA-DR^+^ CD19^+^ CD20^+^ in relation to the total CD19^+^ CD20^+^; (**B**) CD49d+β7^hi^ CD38^+^ CD4^+^ in relation to the total CD4^+^; (**C**) CD103+β7^hi^ CD38^+^ CD8^+^ in relation to the total CD8^+^; and (**D**) CD103+β7^hi^ CD38^+^ TCRγδ^+^ in relation to the total TCRγδ^+^.

## Data Availability

Data is contained within the article.

## Appendix C

**Table A1:** Reagents used in the spectral flow cytometry protocol.

Product	Company	Reference
RBC lysis buffer (10X)	Biolegend	420302
LIVE/DEAD™ Fixable Blue Stain Fluorescence	Invitrogen	L34961
True-Stain Monocyte Blocker™	Biolegend	426102
Brilliant Stain Buffer	BD Biosciences	563794
anti-TCRγδ PerCP-eFluor 710 (clone: B1.1)	Invitrogen	46-9959-42
anti-CX3CR1 in BV711 (clone: 2A9-1)	Biolegend	341630
anti-CCR7 in BV421 (clone: G043H7)	Biolegend	353208
anti-CCR9 in PE/Dazzle (clone: L053E8)	Biolegend	358918
anti-CCR4 in BV750 (clone: 1G1)	BD Biosciences	746980
anti-CD11c in e-Fluor® 450 (clone: 3.9)	Invitrogen	48-0116-42
anti-CD127 in APC-R700 (clone: HIL-7R-M21)	BD Biosciences	565185
anti-CD3 in BV510 (clone: SK7)	Biolegend	344828
anti-CXCR3 in PE/Cy7 (clone: G025H7)	Biolegend	353720
anti-CD20 in Pacific Orange (clone: HI47)	Invitrogen	MHCD2030
anti-CD39 in BUV615 (clone: TU66)	BD Biosciences	751269
anti-PD-1 in BV785 (clone: EH12.2H7)	Biolegend	329930
anti-CD1c in AF647 (clone: L161)	Biolegend	331510
anti-PD-L1 in BV650 (clone: 29E.2A3)	Biolegend	329740
anti-integrin β7 in PerCP/Cy5.5 (clone: FIB27)	Biolegend	121008
anti-CD38 in APC/Fire 810 (clone: HB-7)	Biolegend	356644
anti-CD25 in PE/AF-700 (clone: 3G10)	Invitrogen	MHCD2524
anti-CD123 in SuperBright 436 (clone: 6H6)	Invitrogen	**62-1239-42**
anti-CD14 in Spark Blue 550 (clone: 63D3)	Biolegend	367148
anti-CD45 in PerCP (clone: 2D1)	Biolegend	368506
Anti-CLA in BV605 (HECA-452)	BD Biosciences	563960
anti-CD103 in PE (clone: Ber-ACT8)	Biolegend	350206
anti-CD141 in BB515 (clone: 1A4)	BD Biosciences	566017
anti-CD45RA in BUV395 (clone: 5H9)	BD Biosciences	740315
anti-CD49d in BUV615 (clone: 9F10)	BD Biosciences	751596
anti-CD19 in Spark NIR 685 (clone: HIB19)	Biolegend	302270
anti-CD69 in PE/Cy5 (clone: FN50)	Biolegend	310908
anti-HLA-DR in BUV563 (clone: G46-6)	BD Biosciences	748340
anti-CD56 in BUV737 (clone: NCAM16.2)	BD Biosciences	612766
anti-IgD in BUV480 (clone: IA6-2)	BD Biosciences	566138
anti-CD16 in BUV496 (clone: 3G8)	BD Biosciences	612944
anti-CD8 in BUV805 (clone: SK1)	BD Biosciences	612889
anti-CD4 in cFLUOR^®^ YG584 (clone: SK3)	Cytek	SKU R7-20041
anti-CD27 in APC/Cy7 (clone: M-T271)	Biolegend	356424

## References

[1] Ludvigsson J.F., Leffler D.A., Bai J.C., Biagi F., Fasano A., Green P.H.R., Hadjivassiliou M., Kaukinen K., Kelly C.P., Leonard J.N. (2013). The Oslo definitions for coeliac disease and related terms. Gut.

[2] Anderson R.P., van Heel A.D., Tye-Din A.J., Barnardo M., Salio M., Jewell D.P., Hill A.V.S. (2005). T cells in peripheral blood after gluten challenge in coeliac disease. Gut.

[3] Han A., Newell E.W., Glanville J., Fernandez-Becker N., Khosla C., Chien Y.-H., Davis M.M. (2013). Dietary gluten triggers concomitant activation of CD4+ and CD8+ αβ T cells and γδ T cells in celiac disease. Proc. Natl. Acad. Sci. USA.

[4] López-Palacios N., Pascual V., Castaño M., Bodas A., Fernández-Prieto M., Espino-Paisán L., Martínez-Ojinaga E., Salazar I., Martínez-Curiel R., Rey E. (2018). Evaluation of T cells in blood after a short gluten challenge for coeliac disease diagnosis. Dig. Liver Dis..

[5] Fernández-Bañares F., López-Palacios N., Corzo M., Arau B., Rubio M., Fernández-Prieto M., Tristán E., Pujals M., Farrais S., Horta S. (2021). Activated gut-homing CD8+ T cells for coeliac disease diagnosis on a gluten-free diet. BMC Med..

[6] Christophersen A., Risnes L.F., Dahal-Koirala S., Sollid L.M. (2019). Therapeutic and Diagnostic Implications of T Cell Scarring in Celiac Disease and Beyond. Trends Mol. Med..

[7] Ráki M., Fallang L.-E., Brottveit M., Bergseng E., Quarsten H., Lundin K.E.A., Sollid L.M. (2007). Tetramer visualization of gut-homing gluten-specific T cells in the peripheral blood of celiac disease patients. Proc. Natl. Acad. Sci. USA.

[8] Cook L., Munier C.M.L., Seddiki N., van Bockel D., Ontiveros N., Hardy M.Y., Gillies J.K., Levings M.K., Reid H.H., Petersen J. (2017). Circulating gluten-specific FOXP3^+^ CD39^+^ regulatory T cells have impaired suppressive function in patients with celiac disease. J. Allergy Clin. Immunol..

[9] Tompa A., Faresjö M. (2024). Shift in the B cell subsets between children with type 1 diabetes and/or celiac disease. Clin. Exp. Immunol..

[10] Escudero-Hernández C., Martín Á., de Pedro Andrés R., Fernández-Salazar L., Garrote J.A., Bernardo D., Arranz E. (2020). Circulating Dendritic Cells from Celiac Disease Patients Display a Gut-Homing Profile and are Differentially Modulated by Different Gliadin-Derived Peptides. Mol. Nutr. Food Res..

[11] Sabatino D., Bertrandi E., Maldini C., Pennese F., Proietti F., Corazza G. (1998). Phenotyping of peripheral blood lymphocytes in adult coeliac disease. Immunology.

[12] Cseh Á., Vásárhelyi B., Szalay B., Molnár K., Nagy-Szakál D., Treszl A., Vannay Á., Arató A., Tulassay T., Veres G. (2011). Immune Phenotype of Children with Newly Diagnosed and Gluten-Free Diet-Treated Celiac Disease. Dig. Dis. Sci..

[13] Frisullo G., Nociti V., Iorio R., Patanella A.K., Marti A., Assunta B., Plantone D., Cammarota G., Tonali P.A., Batocchi A.P. (2009). Increased CD4+CD25+Foxp3+ T cells in peripheral blood of celiac disease patients: Correlation with dietary treatment. Hum. Immunol..

[14] Santaguida M.G., Gatto I., Mangino G., Virili C., Stramazzo I., Fallahi P., Antonelli A., Gargiulo P., Romeo G., Centanni M. (2018). Breg Cells in Celiac Disease Isolated or Associated to Hashimoto’s Thyroiditis. Int. J. Endocrinol..

[15] Passerini L., Amodio G., Bassi V., Vitale S., Mottola I., Di Stefano M., Fanti L., Sgaramella P., Ziparo C., Furio S. (2024). IL-10-producing regulatory cells impact on celiac disease evolution. Clin. Immunol..

[16] Baumgaertner P., Sankar M., Herrera F., Benedetti F., Barras D., Thierry A.-C., Dangaj D., Kandalaft L.E., Coukos G., Xenarios I. (2021). Unsupervised Analysis of Flow Cytometry Data in a Clinical Setting Captures Cell Diversity and Allows Population Discovery. Front. Immunol..

[17] Saeys Y., Van Gassen S., Lambrecht B.N. (2016). Computational flow cytometry: Helping to make sense of high-dimensional immunology data. Nat. Rev. Immunol..

[18] Christophersen A., Zühlke S., Lund E.G., Snir O., Dahal-Koirala S., Risnes L.F., Jahnsen J., Lundin K.E.A., Sollid L.M. (2021). Pathogenic T Cells in Celiac Disease Change Phenotype on Gluten Challenge: Implications for T-Cell-Directed Therapies. Adv. Sci..

[19] Morbach H., Eichhorn E.M., Liese J.G., Girschick H.J. (2010). Reference values for B cell subpopulations from infancy to adulthood. Clin. Exp. Immunol..

[20] Perez-Andres M., Paiva B., Nieto W.G., Caraux A., Schmitz A., Almeida J., Vogt R.F., Marti G.E., Rawstron A.C., Van Zelm M.C. (2010). Human peripheral blood B-cell compartments: A crossroad in B-cell traffic. Cytometry B Clin. Cytom..

[21] Knippenberg S., Peelen E., Smolders J., Thewissen M., Menheere P., Tervaert J.W.C., Hupperts R., Damoiseaux J. (2011). Reduction in IL-10 producing B cells (Breg) in multiple sclerosis is accompanied by a reduced naïve/memory Breg ratio during a relapse but not in remission. J. Neuroimmunol..

[22] Christophersen A., Dahal-Koirala S., Chlubnová M., Jahnsen J., Lundin K.E.A., Sollid L.M. (2022). Phenotype-Based Isolation of Antigen-Specific CD4 + T Cells in Autoimmunity: A Study of Celiac Disease. Adv. Sci..

[23] Gómez-Aguililla S., Farrais S., López-Palacios N., Arau B., Senosiain C., Corzo M., Fernandez-Jimenez N., Ruiz-Carnicer A., Fernández-Bañares F., González-García B.P. (2024). Diagnosis of celiac disease on a gluten-free diet: A multicenter prospective quasi-experimental clinical study. medRxiv.

[24] Park L.M., Lannigan J., Jaimes M.C. (2020). OMIP-069: Forty-Color Full Spectrum Flow Cytometry Panel for Deep Immunophenotyping of Major Cell Subsets in Human Peripheral Blood. Cytometry Part. A..

